# Quantitative Formation of Monomeric G‐Quadruplex DNA from Multimeric Structures of c‐Myc Promoter Sequence

**DOI:** 10.1002/cbic.202000159

**Published:** 2020-05-11

**Authors:** Valerie Rauser, Elmar Weinhold

**Affiliations:** ^1^ Institute of Organic Chemistry RWTH Aachen University Landoltweg 1 52074 Aachen Germany

**Keywords:** DNA, G-quadruplexes, monomer, Pu27, size-exclusion chromatography

## Abstract

G‐Quadruplex (G4)‐forming DNA sequences have a tendency to form stable multimeric structures. This can be problematic for studies with synthetic oligodeoxynucleotides. Herein, we describe a method that quantitatively converts multimeric intermolecular structures of the Pu27 sequence from the c‐myc promoter into the desired monomeric G4 by alkaline treatment and refolding.

## Graphical abstract

**A note of caution**: G‐Quadruplex (G4)‐forming DNA sequences have a tendency to form stable multimeric structures that are difficult to denature. This can be problematic for studies with synthetic oligodeoxynucleotides. We describe a method that quantitatively converts multimeric intermolecular structures of Pu27 sequence from c‐myc promoter into desired monomeric G4 by alkaline treatment and refolding.

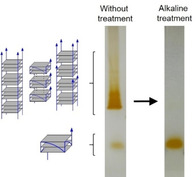

Guanine‐rich DNA sequences are able to adopt three‐dimensional structures called G‐quadruplex (G4) DNA. Four guanines can form a G‐quartet through Hoogsteen base pairing while the *O*6 oxygens are coordinated by cations like K^+^, NH_4_^+^ or Na^+^. At least two of these square planar G‐quartets are involved in the formation of a G4 (Scheme 1).^1^

**Scheme 1:**
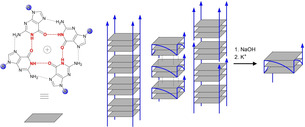
Structure of a G‐quartet with hydrogen bonds between *N*1 and *O*6 highlighted in red and transformation of various multimeric structures into monomeric G4 DNA.

G4‐forming sequences are widespread in the human genome.^2^ They are enriched in promoter regions of many genes including oncogenes like c‐myc,^3^ c‐kit,^4^ VEGF^5^ and bcl‐2^6^ which is of great interest for therapeutic applications like anticancer drug design. The protooncogene c‐myc encodes a protein that is a central regulator of cell growth, differentiation, proliferation and apoptosis.^7^ Overexpression of c‐myc is associated with various cancers like colon, breast, cervix and small cell lung carcinomas.^8^ Expression of c‐myc is mainly controlled by the nuclear hypersensitivity element III1 (NHE III1), that is located −142 to −115 base pairs upstream the P1 promoter.^9^ The template strand of NHE III1 contains the G‐rich sequence Pu27 that is able to form a G4. There is an equilibrium between transcriptionally active forms and G4 structures which function as transcriptional repressor element.7a, 10 When G4s are formed *in vitro* a variety of different intra‐ and intermolecular structures can be obtained.^11^ G‐rich oligodeoxynucleotides (ODN) can not only form monomeric G4 but also bi‐, tri‐ or tetramolecular G4 and other higher order structures like G‐wire.^12^ This can be a major problem when studies with defined monomeric G4 are intended. A common method to obtain defined structures is to modify the sequence by substitution or deletion of guanine nucleotides.^13^ However, this approach can be problematic because the modified G4 might lose its biological relevance. For the native Pu27 sequence for example, a defined structure can be obtained by deleting five G residues and replacing two G residues with T.^3^ The resulting MYC22‐G14T/G23T sequence is quite different compared to the native one.

Trent and co‐workers investigated the influence of strand concentration, annealing process and buffer composition on the distribution of monomer and higher‐order structures of Pu27 after thermal denaturation at 100 °C.^14^ They found that the highest monomer yield is obtained at low K^*+*^ and low strand concentrations. However, even under optimized conditions a significant fraction of the G4 exists in a multimeric form and there is no method described that exclusively forms the biologically relevant monomeric G4 of Pu27.

Because it is also possible to denature duplex DNA^15^ as well as G‐rich ODN^16^ under basic conditions, we were interested to investigate alkaline denaturation for transforming Pu27 ODN into monomeric G4. G‐rich ODN were treated with NaOH followed by neutralization with buffer containing K^+^ ions. For comparison, we performed thermal treatment which is commonly used for unfolding and refolding G4. The efficiency of monomer formation of chemically and thermally treated as well as untreated ODN was analyzed by size‐exclusion chromatography (SEC), which is well suited to investigate the oligomerization state of G4.11a, 17 The chromatogram of Pu27 after DNA synthesis with RP‐HPLC purification shows that the structure of Pu27 is very polymorphic without treatment (Figure 1). A small fraction elutes as monomer with a retention time of around 8 min but most of the ODN elutes earlier, which can be attributed to multimeric structures with higher molecular weight. After thermal treatment at 95 °C for 10 min and cooling to room temperature, the amount of monomer increases but a significant fraction of the ODN is still in its multimeric forms. This demonstrates that thermal denaturation and renaturation by cooling to room temperature is not sufficient to quantitatively produce Pu27 monomers. Only with the chemical treatment all multimeric structures are disrupted and the monomer forms exclusively.

**Figure 1:**
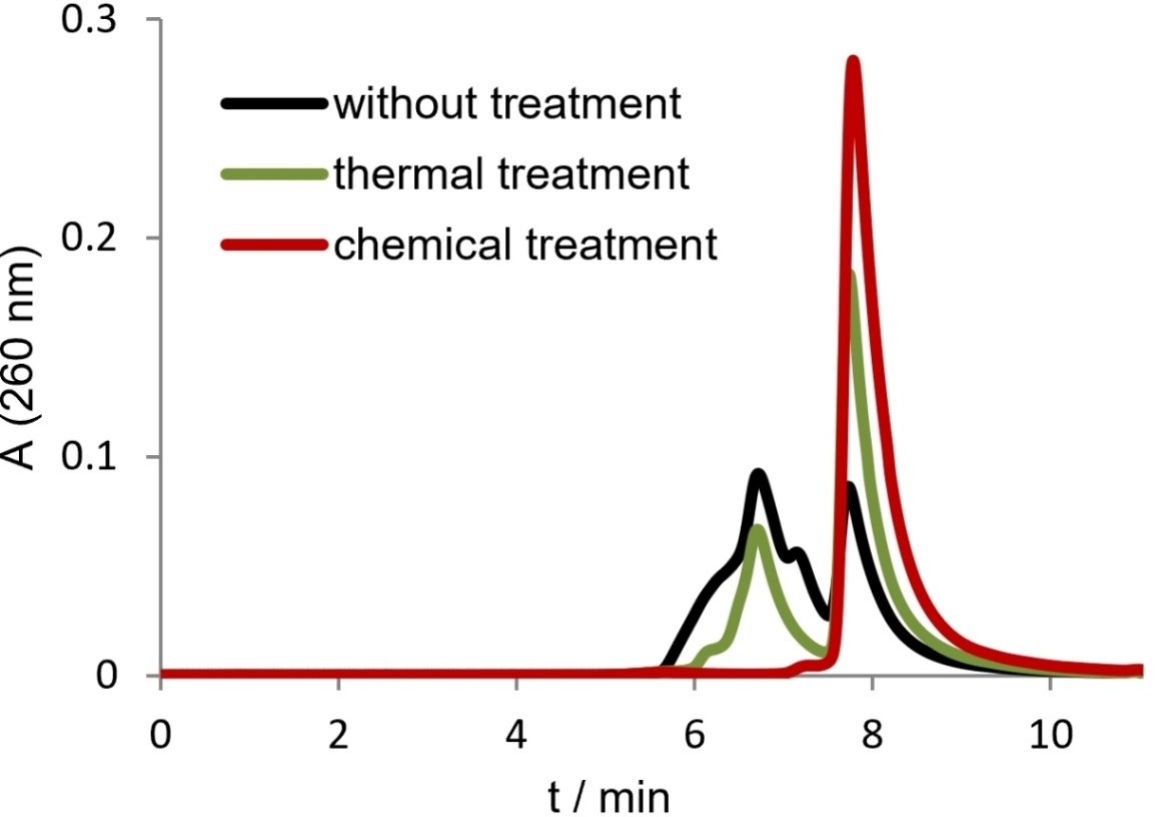
Size‐exclusion chromatography (SEC) of native c‐myc ODN Pu27 without treatment (black), after thermal treatment at 95 °C for 10 min and cooling to room temperature (green) as well as after chemical denaturation with 150 mM NaOH and neutralization with buffer containing K^+^ (red). Very large macromolecules, e. g., bovine thyroglobulin (670 kDa), elute around 5 min.

For the chemical treatment ODN (250 μM) was incubated in an NaOH solution (150 mM) for 5 min at room temperature and then diluted with 50 times the volume of K^+^‐containing buffer (25 mM KH_2_PO_4_, pH 7.0) to neutralize the solution and facilitate monomer formation. Lower NaOH concentrations, even at elevated temperature, did not yield pure monomer (Figures S1 and S2). The disruption of multimeric structures with 150 mM NaOH can be explained by removal of protons from *N*1 of guanine residues, which leads to loss of essential hydrogen bonds for G‐quartet formation (Scheme 1). Loss of *N*1 protons in Pu27 is supported by a change of the UV spectrum upon base treatment which resembles the UV change observed for 2′‐deoxyguanosine (Figure S3).

We also tested the different treatment methods for derivatives of Pu27 (Table 1). In the sequence of Pu22 the first G‐run, which is not involved in monomer G4 formation of Pu27,^18^ is deleted in order to reduce the polymorphism. The second and fourth G‐run contain four G residues as in the native Pu27. In the Myc22 sequence the first G‐run related to Pu27 is deleted, just as in Pu22, and in addition the second and fourth G‐run have three instead of four G in order to reduce polymorphism even more. In the SEC analysis of Pu22 (Figure 2A) and Myc22 (Figure 2B) it becomes obvious that even these sequences form multimeric structures if not treated (black curves). The more the sequence is modified, the more monomers can be observed. Thermal treatment results in less multimers for Pu22 and only monomer is observed for the more modified Myc22 sequence (green curves). After chemical treatment all higher‐order structures also disappear for Pu22 (red curve). The results show that sequence modifications of native Pu27 have a positive influence in reducing the formation of multimeric structures, but if only monomer of Pu27 or slightly modified Pu22 is desired, alkaline treatment is necessary.

**Figure 2:**
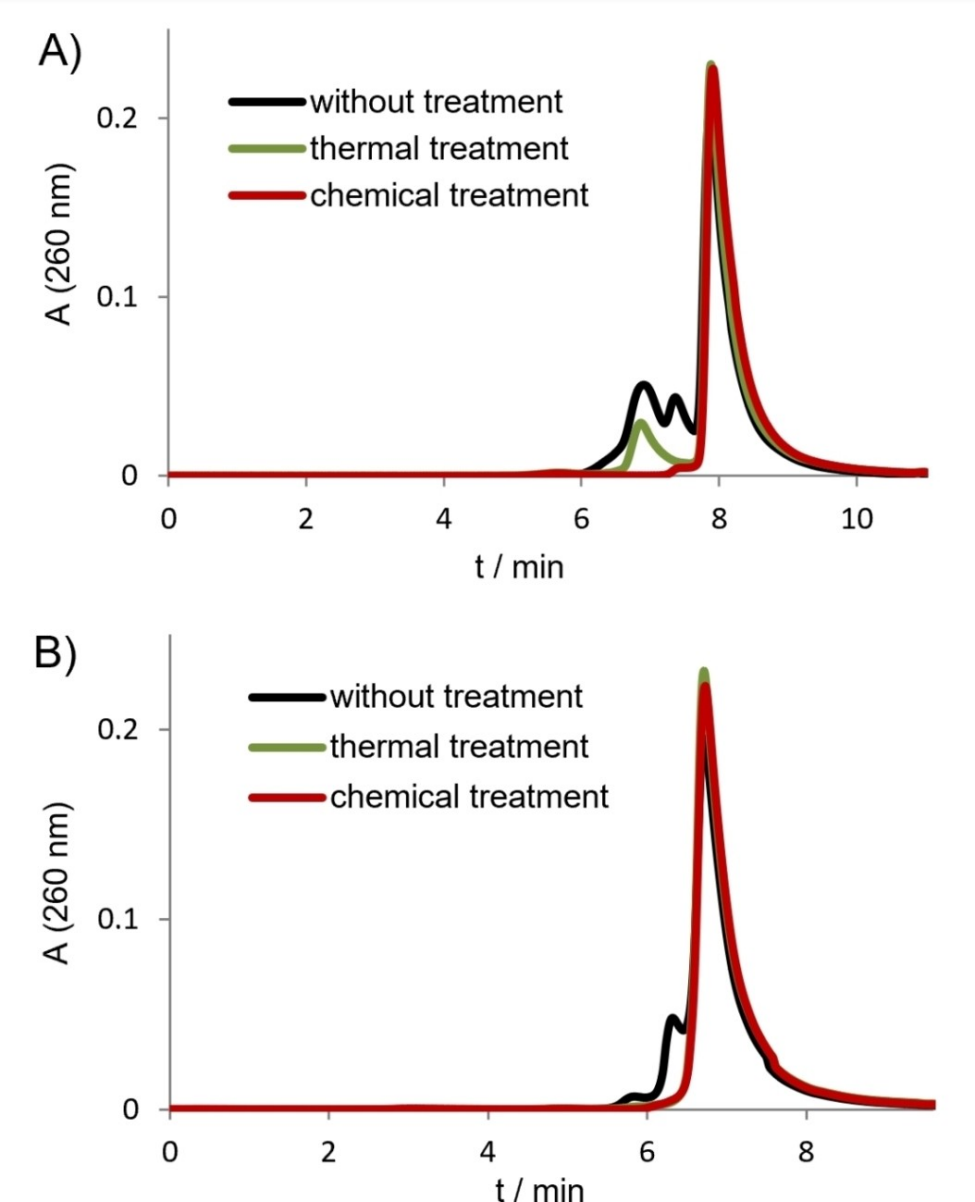
SEC of c‐myc‐derived sequences A) Pu22 and B) Myc22 without treatment (black), after thermal treatment (green) and after chemical treatment (red).

We also examined the treatment methods for the three sequences using polyacrylamide gel electrophoresis (PAGE; Figure 3). On the NuPAGE gel the untreated strands of Pu27 and Pu22 display a fast migrating band and many slower migrating bands that correspond to multimeric structures. After thermal treatment the multimeric structures are still present but the relative amounts of monomer increase. Only denaturation under basic conditions and neutralization with K^+^‐containing buffer converts all multimeric structures into monomers. For Myc22 very low amounts of multimers can be detected without or with thermal treatment and chemical treatment results in pure monomer.

**Figure 3:**
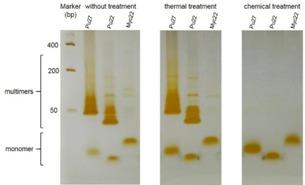
NuPAGE of Pu27, Pu22 and Myc22 without treatment, after thermal treatment and after chemical treatment.

The circular dichroism (CD) spectra of Pu27 before and after the different treatment methods (Figure 4) have a maximum around 260 nm and a minimum around 240 nm what is expected for Pu27 which forms a parallel propeller‐type G4.^18^ These CD spectra show no significant difference although the SEC and NuPAGE analysis demonstrate that there are many different structures. This indicates that CD spectroscopy of Pu27 is not suitable to distinguish between the monomeric and multimeric structures. The CD spectrum of the ODN that was chemically treated also displays the signature of a parallel G4 which demonstrates that the monomer species is not a random coil but a folded G4. It can also be concluded that the multimeric structures have a parallel orientation and even without treatment Pu27 forms G4 structures.

**Figure 4:**
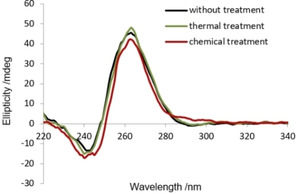
CD spectra of Pu27 without treatment (black), after thermal treatment (green) and after chemical treatment (red).

G4 DNA has another interesting spectroscopic property namely that the absorbance at 295 nm decreases upon melting. We, therefore, investigated the melting behavior of Pu27 and compared the untreated, thermally and chemically treated ODN. The melting temperatures indicated by the inflection points of the melting curves are very similar (Figure 5).

**Figure 5:**
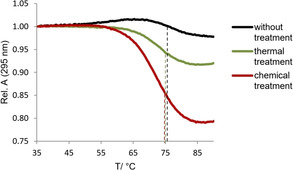
Melting curves of Pu27 without treatment (black), after thermal treatment (green) and after chemical denaturation (red). Dashed lines indicate melting temperatures.

However, a major difference is observed in the amplitude of absorbance change that is highest for the chemically treated and smallest for the untreated ODN. This indicates that the change in absorbance is caused by the melting of the monomeric G4 and that most of the multimeric structures melt above 90 °C. The untreated and thermal treated samples contain a much smaller amount of monomeric G4 and for that reason the change in absorbance is smaller upon heating. The observation that a large fraction of multimeric structures of Pu27 do not melt at 90 °C gives a plausible explanation why the thermal treatment fails to form G4 monomers quantitatively. For the c‐myc‐derived sequences Pu22 and Myc22, which contain less or almost no multimeric structures to begin with, the amplitudes of absorbance change are very similar or almost identical for the samples without, with thermal or with chemical treatment (Figures S4 and S5).

In this work we demonstrated that multimeric G4 DNA structures can be completely converted into monomeric G4 under convenient conditions. By treatment of Pu27 with 150 mM NaOH and neutralization with buffer containing potassium ions monomeric G4 is formed exclusively. We have shown by SEC and PAGE that thermal treatment is not sufficient to disrupt all multimeric structures of Pu27 and its derivative Pu22. In addition, our results demonstrate that the presence of multimeric structures can easily be overlooked when only CD spectra and UV melting points are analyzed. The possibility of having multimeric structures in G4 preparations is rarely discussed in the literature. The presence of higher‐order structures can be problematic for studies with G4 DNA where correct concentrations are needed to obtain binding constants for ligand or protein interactions. In addition, the multimeric structures might behave different than the monomers which could lead to false structure‐function correlations. These problems can be easily overcome by alkaline denaturation followed by refolding in the presence of potassium ions.

## Experimental Section

Oligodeoxynucleotides (ODN) were purchased RP‐HPLC‐purified and lyophilized from Integrated DNA Technologies (IDT; for sequences see Table 1) and dissolved in water. Water was purified using a Milli‐Q purification system and passed through a 0.22 μm filter. Reagents were purchased from Acros Organics, Gerbu, Merck, Serva and Sigma‐Aldrich with analytical grade and used without further purification. The following buffers were prepared: KPP buffer: 25 mM KH_2_PO_4_ (pH 7.0); SEC buffer: 25 mM KH_2_PO_4_, 1 mM NaN_3_ (pH 7.0); NuPAGE gel buffer: 357 mM Bis−Tris (pH 6.5–6.8, NuPAGE running buffer: 50 mM MOPS, 50 mM Tris−X, 5 mM EDTA, 0.1 % SDS (pH 7.6); SDS loading buffer (5x): 250 mM Tris−HCl, 12.5 % SDS, 50 % glycerol, 0.05 % Bromphenol Blue.

**Table 1:** Sequences of G4‐forming oligodeoxynucleotides (ODN).

ODN	Sequence (from 5′ to 3′ direction)
Pu27	TGGGGAGGGTGGGGAGGGTGGGGAAGG
Pu22	TGAGGGTGGGGAGGGTGGGGAA
Myc22	TGAGGGTGGGTAGGGTGGGTGTGAGTG

**Chemical denaturation and refolding of G4 ODN**: An aqueous ODN solution (5 nmol, 5 μL) was supplemented with a solution of NaOH (15 μL, 200 mM) to obtain a final concentration of 150 mM NaOH. The solution was incubated at room temperature for 5 min. Afterwards KPP buffer (980 μL) was added to obtain a neutral pH.

**Thermal denaturation and refolding of G4 ODN**: A solution of ODN in KPP buffer (5 nmol, 1000 μL) in an Eppendorf tube was placed into a heating block (HBT 130 from HLC) at 95 °C for 10 min. After that the tube was removed from the heating block and left outside to cool down to room temperature.

**Size‐exclusion chromatography**: SEC was carried out with a Waters Breeze HPLC system consisting of binary pump system Waters 1525 and Waters 248 Dual λ Absorbance Detector. The system was controlled by the Waters software Empower. For each analysis ODN (350 pmol) was injected onto a SEC column Yarra SEC‐2000 3 μm (300×7.8 mm) purchased from Phenomenex and eluted with SEC buffer at a flow of 1 mL/min. Detection was carried out by UV absorption at 260 and 295 nm.

**Polyacrylamide gel electrophoresis**: Gel electrophoretic analysis was carried out using NuPAGE gels with a 12 % running and 5 % stacking gel. For each lane ODN (35 pmol, 7 μL) was mixed with 5 x SDS loading buffer (1,75 μL) at room temperature which helps to obtain sharper bands. Electrophoresis was carried out at 120 V in a Bio‐Rad mini‐PROTEAN 3 cell for 1 h and DNA bands were visualized by silver staining using a ProteoSilver Silver Stain Kit purchased from Sigma‐Aldrich.

**Circular dichroism spectroscopy**: CD measurements were carried out at Institut Curie, Orsay, France, in cooperation with Dr. Daniela Verga. CD spectra were recorded on a JASCO J‐710 spectropolarimeter connected to a computer. The temperature was set to 20 °C using a peltier temperature controller JASCO PTC‐348WI. A closable quartz cuvette with a 1 cm path length and a volume of 1 mL was used for the measurements. The scans were recorded from 220–500 nm with a sensitivity of 100 mdeg. The data pitch was 1 nm and the scan speed was 200 nm/min with a response time of 1 s. A band width of 1 nm was applied and 4 accumulations were done. ODN were measured at a concentration of 5 μM in KPP buffer and after that the CD spectrum of KPP buffer was subtracted.

**Melting curves**: Melting behaviour of G4 ODN was investigated by measuring the absorption change at 295 nm with a Varian CARY 3E connected to a computer using the program Thermal. The absorption of KPP buffer (500 μL) was measured in a closable quartz cuvette with a 1 cm path length and a total volume of 1 mL and set to zero. Before the melting experiment the buffer was replaced by a solution (500 μL) of (folded) ODN (2.5 nmol, 5 μM) in KPP buffer and melting curves were measured at a temperature range from 35 °C to 90 °C. The data interval was 0.1 °C with a heating rate of 0.3 °C/min. The first derivation of the obtained curves was calculated and the melting temperature obtained by averaging the three lowest values.

## Conflict of interest

The authors declare no conflict of interest.

## Supporting information

As a service to our authors and readers, this journal provides supporting information supplied by the authors. Such materials are peer reviewed and may be re‐organized for online delivery, but are not copy‐edited or typeset. Technical support issues arising from supporting information (other than missing files) should be addressed to the authors.

Supplementary

## References

[1a] J. R. Williamson , Annu. Rev. Biophys. Biomol. Struct. 1994, 23, 703–730; 7919797 10.1146/annurev.bb.23.060194.003415

[1b] M. Gellert , M. N. Lipsett , D. R. Davies , Proc. Natl. Acad. Sci. USA 1962, 48, 2013–2018; 13947099 10.1073/pnas.48.12.2013 PMC221115

[1c] D. Sen , W. Gilbert , Nature 1988, 334, 364–366; 3393228 10.1038/334364a0

[1d] J. T. Davis , Angew. Chem. Int. Ed. 2004, 43, 668–698; 10.1002/anie.200300589 14755695

[2a] A. Bedrat , L. Lacroix , J.-L. Mergny , Nucleic Acids Res. 2016, 44, 1746–1759; 26792894 10.1093/nar/gkw006 PMC4770238

[2b] S. Chambers , G. Marsico , J. M. Boutell , M. D. Antonio , G. P. Smith , S. Balasubramanian , Nat. Biotechnol. 2015, 33, 877–881; 26192317 10.1038/nbt.3295

[2c] N. Maizels , L. T. Gray , PLoS Genet. 2013, 9, e1003468; 23637633 10.1371/journal.pgen.1003468 PMC3630100

[2d] D. Rhodes , H. J. Lipps , Nucleic Acids Res. 2015, 43, 8627–8637. 26350216 10.1093/nar/gkv862 PMC4605312

[3] A. Ambrus , D. Chen , J. Dai , R. A. Jones , D. Yang , Biochemistry 2005, 44, 2048–2058. 15697230 10.1021/bi048242p

[4a] A. T. Phan , V. Kuryavyi , S. Burge , S. Neidle , D. J. Patel , J. Am. Chem. Soc. 2007, 129, 4386–4392; 17362008 10.1021/ja068739h PMC4693632

[4b] S.-T. D. Hsu , P. Varnai , A. Bugaut , A. P. Reszka , S. Neidle , S. Balasubramanian , J. Am. Chem. Soc. 2009, 131, 13399–13409. 19705869 10.1021/ja904007p PMC3055164

[5] D. Sun , K. Guo , J. J. Rusche , L. H. Hurley , Nucleic Acids Res. 2005, 33, 6070–6080. 16239639 10.1093/nar/gki917 PMC1266068

[6] J. Dai , D. Chen , R. A. Jones , L. H. Hurley , D. Yang , Nucleic Acids Res. 2006, 34, 5133–5144. 16998187 10.1093/nar/gkl610 PMC1636422

[7a] A. Siddiqui-Jain , C. L. Grand , D. J. Bearss , L. H. Hurley , Proc. Natl. Acad. Sci. USA 2002, 99, 11593–11598; 12195017 10.1073/pnas.182256799 PMC129314

[7b] T. A. Brooks , L. H. Hurley , Genes Cancer 2010, 1, 641–649; 21113409 10.1177/1947601910377493 PMC2992328

[7c] S. Pelengaris , B. Rudolph , T. Littlewood , Curr. Opin. Genet. Dev. 2000, 10, 100–105. 10679391 10.1016/s0959-437x(99)00046-5

[8a] K. B. Marcu , S. A. Bossone , A. J. Patel , Annu. Rev. Biochem. 1992, 61, 809–860; 1497324 10.1146/annurev.bi.61.070192.004113

[8b] C. A. Spencer , M. Groudine , Adv. Cancer Res. 1991, 56, 1–48. 2028839 10.1016/s0065-230x(08)60476-5

[9a] U. Siebenlist , L. Hennighausen , J. Battey , P. Leder , Cell 1984, 37, 381–391; 6327064 10.1016/0092-8674(84)90368-4

[9b] M. Cooney , G. Czernuszewicz , E. H. Postel , S. J. Flint , M. E. Hogan , Science 1988, 241, 456–459; 3293213 10.1126/science.3293213

[9c] T. L. Davis , A. B. Firulli , A. J. Kinniburgh , Proc. Natl. Acad. Sci. USA 1989, 86, 9682–9686; 2690070 10.1073/pnas.86.24.9682 PMC298565

[9d] I. Wierstra , J. Alves , Adv. Cancer Res. 2008, 99, 113–333. 18037408 10.1016/S0065-230X(07)99004-1

[10] V. Gonzalez , L. H. Hurley , Annu. Rev. Pharmacol. Toxicol. 2010, 50, 111–129. 19922264 10.1146/annurev.pharmtox.48.113006.094649

[11a] M. C. Miller , H. T. Le , W. L. Dean , P. A. Holt , J. B. Chaires , J. O. Trent , Org. Biomol. Chem. 2011, 9, 7633–7637; 21938285 10.1039/c1ob05891f PMC3962748

[11b] A. N. Lane , J. B. Chaires , R. D. Gray , J. O. Trent , Nucleic Acids Res. 2008, 36, 5482–5515. 18718931 10.1093/nar/gkn517 PMC2553573

[12a] N. Smargiasso , F. Rosu , W. Hsia , P. Colson , E. S. Baker , M. T. Bowers , E. De Pauw , V. Gabelica , J. Am. Chem. Soc. 2008, 130, 10208–10216; 18627159 10.1021/ja801535e

[12b] P. Tóthová , P. Krafčíková , V. Víglaský , Biochemistry 2014, 53, 7013–7027; 25347520 10.1021/bi500773c

[12c] W. I. Sundquist , A. Klug , Nature 1989, 342, 825–829; 2601741 10.1038/342825a0

[12d] T. C. Marsh , J. Vesenka , E. Henderson , Nucleic Acids Res. 1995, 23, 696–700. 7899091 10.1093/nar/23.4.696 PMC306740

[13a] D. Yang , K. Okamoto , Future Med. Chem. 2010, 2, 619–646; 20563318 10.4155/fmc.09.172 PMC2886307

[13b] R. I. Mathad , E. Hatzakis , J. Dai , D. Yang , Nucleic Acids Res. 2011, 39, 9023–9033. 21795379 10.1093/nar/gkr612 PMC3203601

[14] H. T. Le , M. C. Miller , R. Buscaglia , W. L. Dean , P. A. Holt , J. B. Chaires , J. O. Trent , Org. Biomol. Chem. 2012, 10, 9393–9404. 23108607 10.1039/c2ob26504d PMC3501587

[15a] P. Ehrlich , P. Doty , J. Am. Chem. Soc. 1958, 80, 4251–4255;

[15b] N. Borovok , T. Molotsky , J. Ghabboun , D. Porath , A. Kotlyar , Anal. Biochem. 2008, 374, 71–78. 17996714 10.1016/j.ab.2007.10.017

[16a] P. B. Arimondo , J.-F. Riou , J.-L. Mergny , J. Tazi , J.-S. Sun , T. Garestier , C. Hélène , Nucleic Acids Res. 2000, 28, 4832–4838; 11121473 10.1093/nar/28.24.4832 PMC115246

[16b] S. Lyonnais , C. Hounsou , M.-P. Teulade-Fichou , J. Jeusset , E. Le Cam , G. Mirambeau , Nucleic Acids Res. 2002, 30, 5276–5283; 12466553 10.1093/nar/gkf644 PMC137959

[16c] P. L. T. Tran , A. De Cian , J. Gros , R. Moriyama , J.-L. Mergny , Top. Curr. Chem. 2013, 330, 243–274; 22752578 10.1007/128_2012_334

[16d] J. Zhou , F. Rosu , S. Amrane , D. N. Korkut , V. Gabelica , J. L. Mergny , Methods 2014, 67, 159–168. 24434505 10.1016/j.ymeth.2014.01.004

[17] E. Largy , J.-L. Mergny , Nucleic Acids Res. 2014, 42, e149. 25143531 10.1093/nar/gku751 PMC4231728

[18a] A. T. Phan , Y. S. Modi , D. J. Patel , J. Am. Chem. Soc. 2004, 126, 8710–8716; 15250723 10.1021/ja048805k PMC4692381

[18b] D. Yang , L. H. Hurley , Nucleosides Nucleotides Nucleic Acids 2006, 25, 951–968. 16901825 10.1080/15257770600809913

