# Supplementary material for: Quantitative Formation of Monomeric G‐Quadruplex DNA from Multimeric Structures of c‐Myc Promoter Sequence
Source: Chembiochem. 2020 May 11;21(17):2445–8. doi: 10.1002/cbic.202000159 (PMC7496815; doi:10.1002/cbic.202000159)
Supplement: Supplementary file 1 — Supplementary [file CBIC-21-2445-s001.pdf]

ChemBioChem

Supporting Information

Quantitative Formation of Monomeric G-Quadruplex DNA from Multimeric Structures of c-Myc Promoter Sequence

Valerie Rauser and Elmar Weinhold*© 2020 The Authors. Published by Wiley-VCH Verlag GmbH & Co. KGaA.
This is an open access article under the terms of the Creative Commons Attribution License, which permits use, distribution and reproduction in any medium, provided the original work is properly cited.

Table of Contents

Optimization of alkaline denaturation 1

UV spectra under neutral and basic conditions 2

Melting curves of Pu22 and Myc22 4

Reference... 5

Optimization of alkaline denaturation

Alkaline denaturation was investigated using different NaOH concentrations. An aqueous solution of Pu27 ODN (1 nmol, 1 µL) was mixed with an equal volume of NaOH (200 mM, 300 mM and 400 mM) to obtain a final concentrations of 100, 150 and 200 mM. The samples were incubated at room temperature for 5 min followed by addition and of a large excess of KPP buffer (198 µL). Subsequent analysis (70 µL, 350 pmol) by size exclusion chromatography (column Yarra SEC-2000 3 µm, 300 x 7.8 mm, isocratic elution with SEC buffer and a flow of 1 mL/min, UV detection at 260 and 295 nm) revealed complete monomer formation for incubations with 150 and 200 mM NaOH while incubation with 100 mM NaOH gave a mixture of monomeric and multimeric structures indicating inefficient denaturation (Fig. S1).

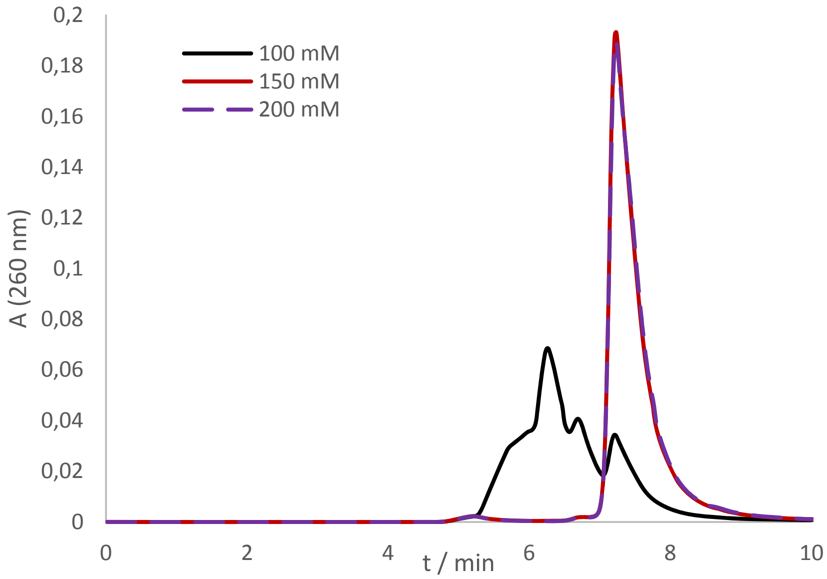

Figure S1. Size exclusion chromatography (SEC) of native ODN Pu27 after alkaline treatment with 100 mM (black), 150 mM (red) and 200 mM (purple, dashed) NaOH and neutralization with excess of K^+-containing buffer.

In addition, we investigated the influence of increased incubation temperature on monomer formation upon treatment with 100 mM NaOH. Pu27 in NaOH (100 mM) was incubated at 55 °C for 10 min and after cooling to room temperature a large excess of KPP buffer was added as before. Analysis by size exclusion chromatography indicates that heating the sample helps to denature multimeric structures and more monomer is formed at 55 °C than at room temperature (Fig. S2). Nevertheless, a significant amount of multimeric structures remains at 55 °C. This shows that a combination of low NaOH concentration (100 mM) and a gentle thermal treatment is not sufficient to produce the monomer exclusively. Therefore, all further alkaline treatments of Pu27 (as well as of Pu22 and Myc22) were performed at 150 mM NaOH and room temperature.

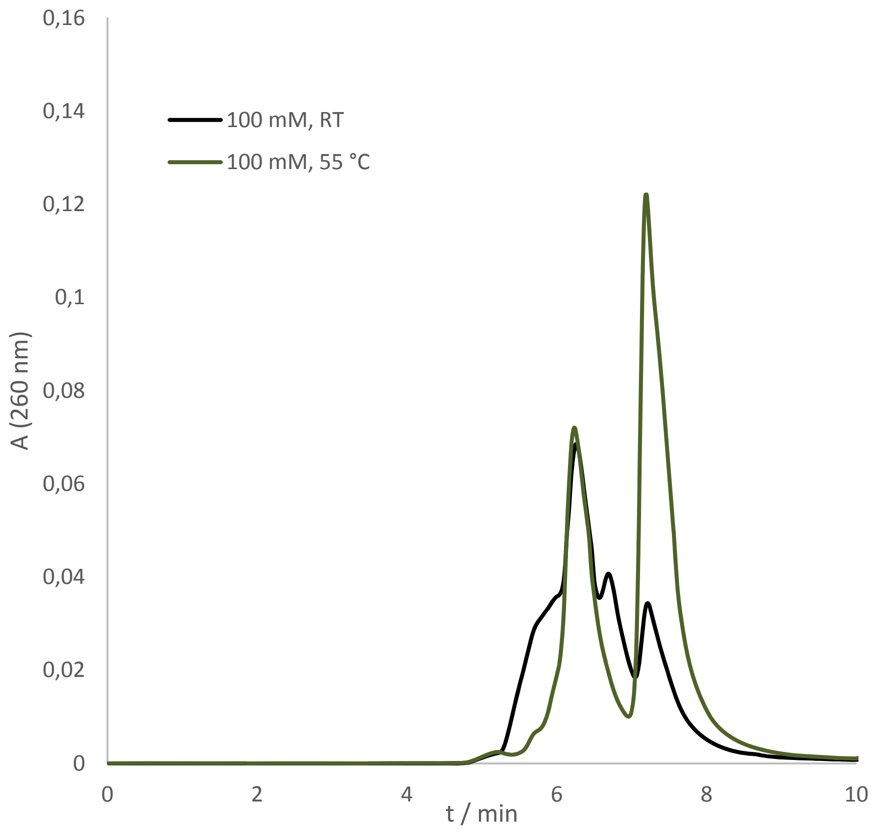

Figure S2. Size exclusion chromatography (SEC) of the native ODN Pu27 after alkaline treatment with 100 mM NaOH at 55 °C for 10 min (green) or at room temperature (RT) for 5 min (black, same as in Fig. S1) and neutralization with excess of K^+-containing buffer.

UV spectra under neutral and basic conditions

Unfolding of the multimeric Pu27 structures can be explained by loss of hydrogen bonds between *N*1 and *O*6 in the G-quartets. Under strongly basic conditions (150 mM NaOH, pH > 13) the *N*1 position of guanines is deprotonated,[1] which abolishes its function as hydrogen bond donor. Evidence for deprotonation was obtained by comparing the UV spectra of 2'-deoxyguanosine (dG) and Pu27 in KPP buffer and NaOH solution (Fig. S3).

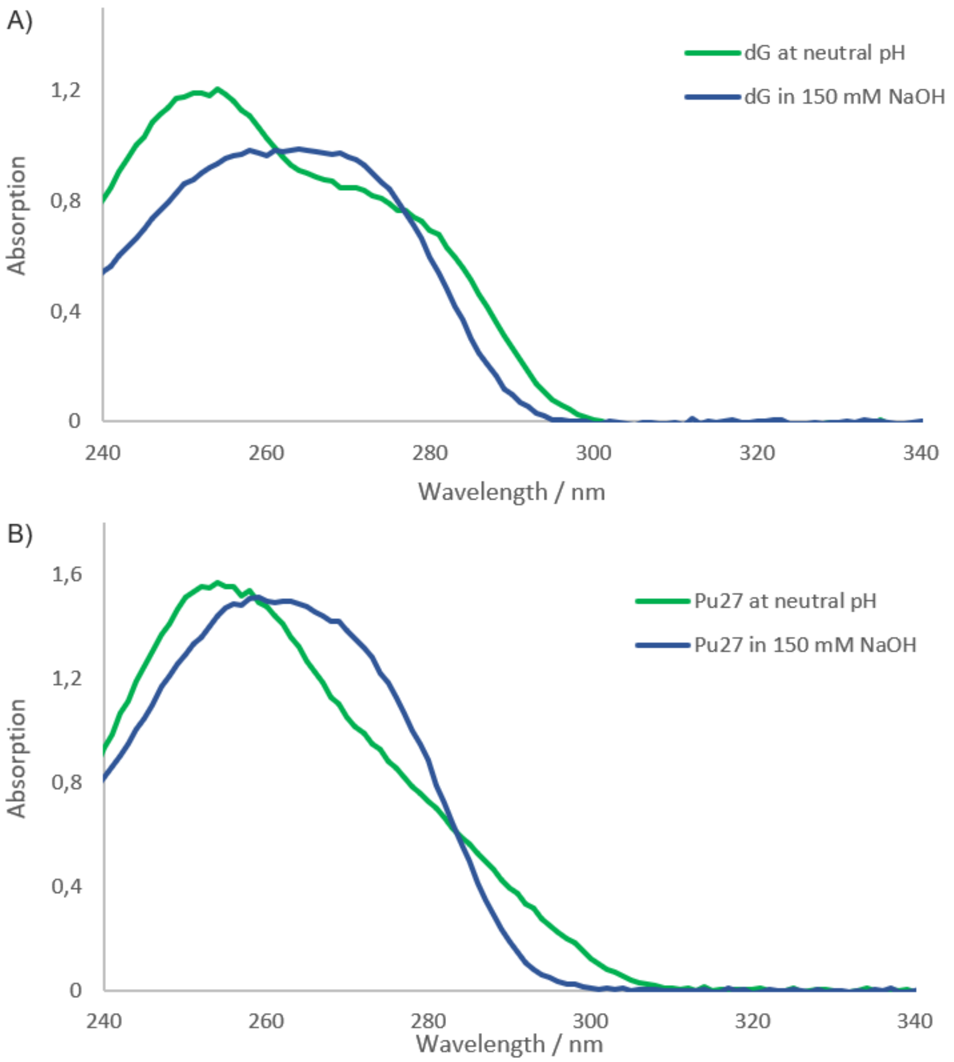

Figure S3. A) UV spectra of 2'-deoxyguanosine (dG) in KPP buffer (green) and in 150 mM NaOH (blue) and B) of untreated Pu27 in KPP buffer (green) and Pu27 in 150 mM NaOH (blue).

UV spectra were measured with a Varian CARY 3E connected to a computer using the program Scan. The spectra were recorded at 25 °C from 240 to 340 nm with a data interval of 1 nm. The baseline of pure KPP buffer or 150 mM NaOH solution was recorded in a quartz cuvette with a 1 cm path length. After that, dG (50 nmol, 100 µM final concentration) and Pu27 (2.5 nmol, 5 µM final concentration) were dissolved in KPP buffer (500 µL) or 150 mM NaOH (500 µL) to measure the UV spectra. The UV spectrum of dG at neutral pH shows a maximum at 254 nm and an absorption shoulder around 280 nm (Fig. S3A). In 150 mM NaOH the absorption maximum shifts towards higher wavelengths but decreases sharply above 280 nm. The change in the UV spectrum is explained by deprotonation at *N*1 of dG. The UV spectrum of Pu27 without treatment in KPP buffer also shows a maximum around 254 nm (Fig. S3B). At basic pH (150 mM NaOH) the UV spectrum shifts towards higher wavelengths and resembles that of dG in NaOH. This indicates that the guanine residues of Pu27 are deprotonated upon NaOH treatment.

Melting curves of Pu22 and Myc22

The melting curves of Pu22 show the typical melting behavior of a G4 at 295 nm (Fig. S4). The melting temperatures are very similar but the melting curve of Pu22 without treatment has a slightly lower amplitude compared to the other two curves obtained after thermal or chemical treatment. This is in line with the observation that even without treatment a large fraction of Pu22 is already in its monomeric form as observed by SEC analysis (Fig. 2A). The melting curves after thermal and chemical treatment show identical amplitudes. The three melting curves of Pu22 are more similar to each other than those of Pu27 (compare Fig. 5) which reflects the much lower amount of multimeric structures in untreated Pu22.

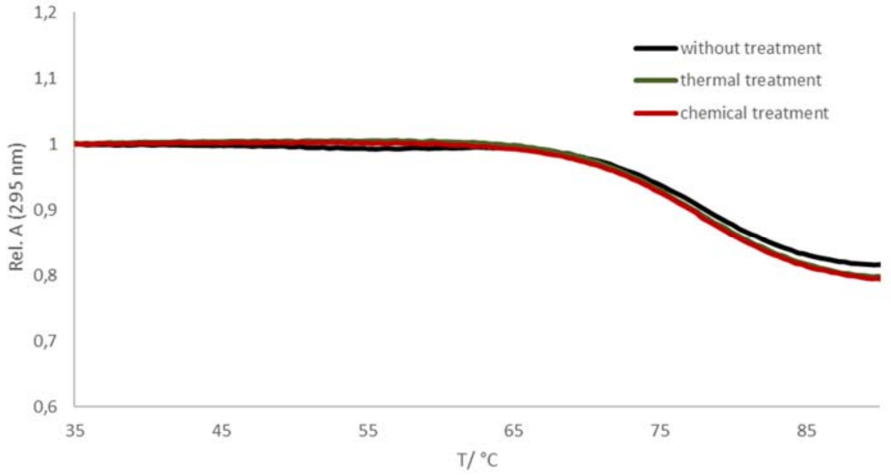

Figure S4. Melting curves of Pu22 without treatment (black), after thermal treatment (green) and after chemical treatment (red).

The melting curves of Myc22 (Fig. S5) show almost identical amplitudes. This corresponds to the high similarity of the size exclusion chromatograms where the Myc22 monomer is detected almost exclusively (Fig. 2B).

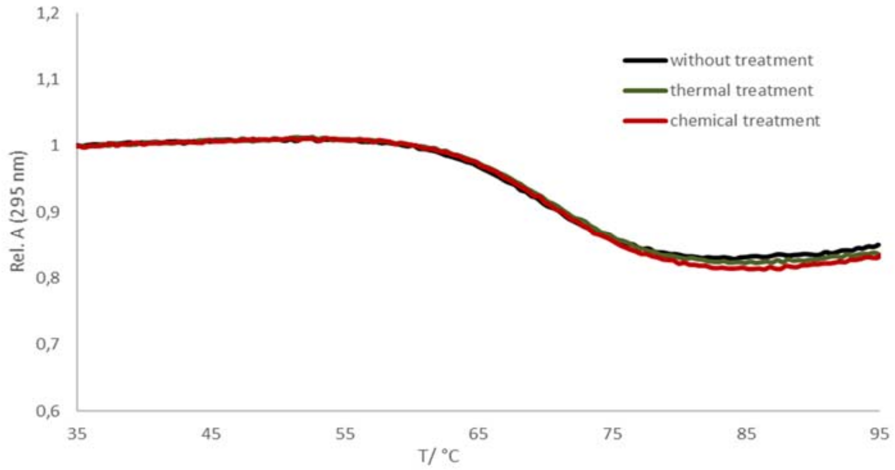

Figure S5. Melting curves of Myc22 without treatment (black), after thermal treatment (green) and after chemical treatment (red).

Reference

1 R. L. Lundblad, F. M. Macdonald, *Handbook of Biochemistry and Molecular Biology*, Fourth edition, CRC Press, 2010.
